# John William Newby

**Published:** 1823-08

**Authors:** 


### OBITUARY.

---

On Wednesday, the 4th of June, John William Newby, Esq. apothecary, of Poland-street.

The circumstances attending this gentleman's death were of a very peculiar and melancholy nature; and we feel much obliged to Dr. Nelson for the following account of the case, which cannot fail of being highly interesting to our readers.

On Saturday, the 31st of May, Mr. Newby was exposed to a heavy rain; on the following day he opened the body of a child which died of enteritis, having also, as I was informed, erysipelas of the abdomen. On the Monday and Tuesday he was, as usual, occupied in his profession, but complained of great languor and depression. On the evening of Wednesday I was requested to see him. At that time he complained of head-ache, general pains of his limbs, heat, nausea, and constipated bowels. His pulse was frequent, but neither hard nor full; the tongue was white. He showed me a pustule on the back of the left thumb, exactly resembling the small-pox, but did not in any way account for its appearance, nor even mention the circumstance of having opened the body of the child on the preceding Sunday. He had slight pain in the axilla, without tumor, or any appearance of inflamed lymphatics on the arm. He had taken a purgative and diaphoretics, by the advice of his friend Mr. Barry, which we directed to be continued. The evacuations from the bowels were very offensive. On Thursday he seemed somewhat better, and continued so till the evening of Friday. The thumb gave very little uneasiness, and the pain in the axilla was much diminished. During the day he took more nourishment, and a little wine and water.

Saturday.—He had passed a restless night, and complained of a deep-seated pain in the left breast, which assumed a light pink tinge, and the axilla and arm became more uneasy; the thumb being nearly well. I requested that Mr. Copeland might be sent for, to give us his advice and assistance in the management of the diseased part. The pulse increased in frequency. He took very little nourishment or medicine during the day, but had an opiate at bed-time.

Sunday.—He had some sleep in the early part of the night, but afterwards excessive irritability, with slight delirium. The inflammation of the breast had extended, and was surrounded with a deeper red margin, of about one-eighth of an inch in breadth; the pulse was about 108, more feeble; the tongue had become dry and brown. He had during this day Infus. cuspariæ, with acid. muriat. and opium, given in considerable doses every four hours, wine, &c. In the evening he was visited by Dr. Hooper.

Monday.—He passed a very restless night, though the opium had considerably lessened the irritability. The pain of the head was greater, and that of the left breast much increased; the tumor had extended from the sternum to the scapula, and from the clavicle to the left hypochondrium; the heat of the skin was much greater; the tongue was dry, and brown; the pulse 110. He had had two fetid stools. The medicines were continued. He was very restless during the day.

Tuesday.—The arm had swelled during the night, and the tumor of the breast appeared to contain a considerable quantity of effused serum, and was of a brownish yellow colour; the symptoms nearly as on Monday. Decoct. cinchonæ, with acid sulph. dilut., opium, and wine, were directed to be given.

Wednesday.—Had passed a restless night; all the symptoms were aggravated; and he died about twelve.

It is worthy of remark that, during Mr. Newby's illness, Mr. Jackson, his assistant, had an inflammation of the fauces, of an erysipelatous appearance, which terminated in suppuration of the tonsil. His pupil had an attack of low fever, which continued about a week. The house-maid was severely affected with cynanche tonsillaris, which terminated by resolution. The nurse had a slight attack of pyrexia, with pain and stiffness of the back of the neck; on account of which she went home for a day or two; but, returning to the house, she was atacked with erysipelas phlegmonodes, which proved fatal. Another woman, who assisted in the room after Mr. Newby's death, had also the erysipelas phlegmonodes, but recovered.

Was the disease which destroyed Mr. N. erysipelas produced by inoculation, affecting the cellular substance of the breast and parts adjoining? Did the five cases which occurred during his disease and after his death, arise from erysipelatous contagion?

### NOTICES TO CORRESPONDENTS.

